# Exploring multilevel determinants of nursing documentation quality in suicide care: A qualitative Study in Iranian hospitals

**DOI:** 10.1371/journal.pone.0356460

**Published:** 2026-09-02

**Authors:** Ali Reza Yusefi, Omolbanin Atashbahar, Ali Asghar Kheirkhah Vakilabad, Hedyeh Askarpour, Jamshid Bahmeai

**Affiliations:** 1 Student Research Committee, Sirjan School of Medical Sciences, Sirjan, Iran; 2 Department of Public Health, Sirjan School of Medical Sciences, Sirjan, Iran; 3 Assistant Professor of Internal Medicine, Department of Internal Medicine, School of Medicine, Imam Khomeini Hospital, Jiroft University of Medical Sciences, Jiroft, Iran; 4 School of Medicine, Jiroft University of Medical Sciences, Jiroft, Iran; 5 Department of Public Health, Behbahan Faculty of Medical Sciences, Behbahan, Iran; North-West University, SOUTH AFRICA

## Abstract

**Background:**

High-quality nursing documentation is crucial for patient safety, continuity of care, and legal accountability, particularly in suicide-related care. Despite its importance, documentation practices in such sensitive contexts are influenced by multiple interacting factors. This study aimed to explore the determinants of high-quality nursing documentation in suicide cases from the perspectives of nurses and clinical supervisors in Iran.

**Methods:**

A qualitative study using conventional content analysis was conducted in 2024 across multiple hospitals in Iran. Purposive sampling with maximum variation was used to recruit 25 participants, including registered nurses, head nurses, and clinical supervisors involved in suicide care. Maximum variation was ensured across hospital types (general hospitals with emergency departments and specialized psychiatric units), clinical roles, years of experience, and geographical regions. Data were collected through in-depth, semi-structured interviews, transcribed verbatim, and analyzed following Graneheim and Lundman’s approach. MAXQDA 2022 software was used for data management. Trustworthiness was ensured using Lincoln and Guba’s criteria.

**Results:**

Analysis revealed eight main interrelated themes encompassing organizational, professional, interpersonal, and systemic determinants of nursing documentation quality. These included Work Environment and Organizational Support, Professional Knowledge and Competence, Training and Continuing Education, Communication and Collaboration, Staffing and Workload Balance, Motivation and Accountability, Use of Technology and Resources, and Supervision and Feedback. Together, these factors acted as both facilitators and barriers, shaping the accuracy, completeness, and consistency of nursing documentation in suicide- related care. Beyond common documentation factors, the findings highlighted suicide-specific challenges, including the need for precise recording of fluctuating suicide risk, documentation of sensitive patient disclosures, ethical tensions between confidentiality and safety, and the impact of high-stress, time-critical decision-making on documentation accuracy.

**Conclusion:**

The findings indicate that improving documentation requires a multifaceted approach that strengthens professional capacity, fosters supportive organizational cultures, optimizes workload and resources, leverages appropriate technologies, and ensures constructive supervision. Particularly in suicide care, where documentation has critical clinical and legal implications, targeted strategies are needed to support accurate recording of risk, patient behavior, and interdisciplinary decision-making. Addressing these determinants through targeted training, supportive organizational policies, adequate staffing, and structured supervision can enhance patient safety, continuity of care, and the effectiveness of suicide prevention efforts.

## Introduction

Nursing documentation is a fundamental component of professional nursing practice, encompassing the structured, standardized, and chronological recording of patient assessments, nursing interventions, care plans, and outcomes in clinical records [1]. High-quality documentation serves multiple purposes; it ensures continuity of care, facilitates communication among healthcare team members, supports legal accountability, and serves as important evidence when legal issues arise, supports clinical decision-making, and enables quality improvement initiatives. In mental health care, particularly in the management of patients with suicidal behaviors, the quality of nursing documentation is especially important [2–4]. Accurate and comprehensive records not only allow for timely and effective interventions but also provide valuable data for evaluating risk factors, monitoring patient progress, and guiding preventive strategies [3,4].

Suicide is a significant public health issue worldwide and remains a leading cause of death across multiple age groups [5]. Nurses are often at the forefront of patient care and play a critical role in identifying suicide risk, implementing preventive measures, and providing psychosocial support [6]. Effective nursing documentation in suicide cases is essential to ensure that patients receive consistent, evidence-based care and that mental health interventions are properly evaluated and refined over time [7].

Despite its recognized importance, nursing documentation in suicide cases has been shown to have deficiencies [8]. Studies indicate that records may be incomplete, inconsistent, or lacking critical details regarding patient assessments, risk factors, and interventions [8]. Multiple factors contribute to this challenge. Heavy workloads, time constraints, insufficient training in mental health documentation, emotional stress associated with managing suicidal patients, and the lack of standardized documentation protocols can all negatively affect the completeness and accuracy of nursing records [9,10]. Consequently, poor-quality documentation may compromise patient safety, hinder clinical decision-making, limit the effectiveness of suicide prevention programs, and reduce the quality of mental health research data [7,11]. Within the Iranian healthcare context, this issue is particularly relevant in hospital settings where mental health services are integrated into general healthcare units, as nurses may face competing demands, limited resources, and inadequate support when caring for patients with complex psychosocial needs [12]. Understanding the multifaceted factors that influence documentation quality is therefore critical to improving both clinical outcomes and organizational practices [13].

Improving the quality of nursing documentation, particularly in suicide cases, is not only a professional and ethical obligation but also a strategic priority for healthcare systems [14]. Comprehensive and accurate documentation enhances patient safety, ensures continuity of care, informs clinical decision-making, and provides a foundation for evidence-based interventions [15,16]. Moreover, well-documented patient records contribute to research and policy development, allowing health authorities to evaluate mental health programs, identify systemic gaps, and implement targeted improvements [17].

In the context of Iran, where suicide rates have shown concerning trends in certain populations [18], the role of nurses in suicide prevention is particularly critical [19]. However, suicide in Iran is embedded in a complex socio-cultural, religious, and legal context [20]. Suicide is strongly stigmatized because of religious beliefs and may also be associated with legal, familial, and social consequences, even when suicide attempts are not formally prosecuted [21]. These sensitivities can influence how patients disclose suicidal intent and how healthcare professionals document such information [22]. Nurses may experience professional pressure to document cautiously, avoid stigmatizing labels, or omit sensitive details because of concerns about legal repercussions, family reactions, or institutional scrutiny [23]. This context may lead to under-documentation, ambiguity, or defensive recording practices, ultimately affecting the accuracy and completeness of nursing records in suicide care.

Within Iranian hospital settings, nurses caring for patients following suicide attempts may encounter uncertainty regarding the documentation of suicide risk assessments, patients’ disclosures, family concerns, and psychosocial interventions. The sensitive nature of suicide-related information, together with variations in organizational practices, can contribute to inconsistencies in nursing documentation [13]. Despite the recognized importance of accurate documentation, little qualitative evidence is available to explain how nurses experience these challenges or which contextual factors influence documentation practices in Iranian hospitals. These practical and knowledge gaps provided the primary motivation for conducting the present study.

Although nursing documentation has been widely studied in general clinical settings, few studies have specifically examined documentation in suicide care using qualitative methods. Most existing research has relied on quantitative record audits that identify documentation deficiencies but provide limited insight into why these challenges occur in everyday clinical practice. Furthermore, evidence from low- and middle-income countries, including Iran, remains scarce, highlighting the need for context-specific research to inform locally relevant interventions.

To address these gaps, the present study explored the factors influencing the quality of nursing documentation in suicide cases using a qualitative approach in Iranian hospitals. By capturing nurses’ experiences, perceptions, and challenges, the study sought to identify the individual, interpersonal, organizational, and contextual factors that shape documentation practices. The findings may provide context-specific evidence to support the development of documentation standards, educational interventions, organizational policies, and patient safety strategies aimed at improving suicide care in Iranian hospitals.

## Methods

### Study design and Setting

This qualitative study employed a conventional content analysis approach in accordance with Graneheim and Lundman’s (2004) approach to qualitative content analysis [24]. This method is well suited for exploring complex and under-investigated aspects of nursing documentation in sensitive clinical situations, such as suicide cases. The primary aim was to identify and interpret factors influencing the quality of nursing documentation, with attention to both facilitating and hindering conditions as perceived by nurses and other key stakeholders. The study was conducted in multiple hospitals affiliated with medical universities across Iran, including Tehran, Shiraz, Isfahan, Mashhad, and Kerman, from February 8, 2024, to December 9, 2024. These hospitals were selected because of their central role in providing psychiatric and emergency care, their diverse organizational structures, and their ongoing initiatives to improve mental health nursing practices. The study settings included both general hospitals with active emergency departments managing acute suicide attempts and specialized psychiatric units providing ongoing mental health care. This variation allowed the study to capture differences in documentation practices between high-pressure emergency contexts and more structured psychiatric care environments. Conducting the study across several institutions enabled richer data collection by capturing a wide spectrum of professional experiences and contextual variations.

### Participants

Participants were recruited through purposive sampling guided by the principle of maximum variation to ensure diverse perspectives. The study population included registered nurses, head nurses, and clinical supervisors who were directly involved in the care of patients following suicide attempts. Efforts were made to include participants with a range of ages, genders, years of clinical experience, hospital departments, and educational backgrounds to capture comprehensive insights into factors influencing documentation quality. Maximum variation also included diversity in clinical settings (emergency departments versus psychiatric wards), enabling comparison of documentation challenges across different care environments. Sampling continued until data saturation was achieved, meaning that no new codes or themes emerged from additional interviews. A total of 25 participants were ultimately included in the study.

### Inclusion and exclusion criteria

Inclusion criteria comprised nurses with at least two years of clinical experience in mental health or emergency departments, direct involvement in the care of patients following suicide attempts, and willingness to participate in an audio-recorded interview. Participants were also required to provide written informed consent. Nurses were excluded if they had not been actively involved in the care of patients following suicide attempts during the previous six months, were unable or unwilling to provide informed consent, or withdrew from participation during the interview process.

### Pilot testing of the interview guide

The semi-structured interview guide was developed by the research team based on an in-depth review of both international and national literature on nursing documentation and suicide care (see Supplementary File 1). The preliminary guide was pilot-tested with three participants, including one senior nurse, one clinical supervisor, and one psychiatric nurse who were not included in the main study. Feedback focused on question clarity, comprehensibility, and flow, leading to refinements such as simplifying technical terms (e.g., “risk assessment documentation” was rephrased as “recording patient risk factors”) and reordering questions to improve logical progression. Probing prompts, such as “Can you describe an example of when documentation affected patient care?”, were added to elicit richer responses. The final guide contained open-ended and probing questions, including: “What factors facilitate or hinder high-quality nursing documentation in suicide cases?”, “Can you describe challenges you face when documenting care for suicidal patients?”, and “What changes could improve documentation practices in your unit?”

### Data collection

Data were collected through in-depth, semi-structured interviews conducted either face-to-face in a quiet office within the hospital or virtually via secure online platforms, depending on participants’ preferences and logistical considerations. Each interview lasted 40–65 minutes and was conducted in Persian by members of the research team experienced in qualitative methods and familiar with the cultural and organizational context of nursing in Iran. To minimize translation bias, all interviews were first transcribed verbatim in Persian and then independently translated into English by two bilingual researchers. The translated transcripts were compared, and any discrepancies were resolved through discussion and consensus. In addition, the final English version was reviewed by a senior researcher fluent in both languages to ensure conceptual equivalence rather than a literal translation. Before the interviews, participants received an information sheet explaining study objectives, voluntary participation, confidentiality, and their right to withdraw at any time. Written informed consent was obtained, and verbal confirmation was recorded at the start of each session. All interviews were audio-recorded, transcribed verbatim, and cross-checked for accuracy. Field notes and reflexive memos were documented to capture non-verbal cues, contextual observations, and preliminary analytical insights. In addition, structured debriefing sessions were held regularly among the research team throughout data collection. After each interview, researchers met to discuss initial impressions, emerging patterns, and potential biases in data interpretation. These discussions were also used to refine probing strategies and ensure consistency in data collection and analysis. Interviews were iterative, allowing emerging themes to guide subsequent questioning. Data saturation was determined through an iterative process in which emerging codes from each interview were continuously compared with the existing codebook. A saturation grid was used to monitor the emergence of new codes, and saturation was considered achieved when two consecutive interviews yielded no new codes or conceptual insights.

### Data analysis

Data were analyzed using an inductive conventional content analysis approach as described by Graneheim and Lundman [24]. Transcripts were read multiple times to gain familiarity with participants’ narratives. Meaning units, defined as phrases or sentences conveying a single idea, were identified, condensed, and coded. Conceptually similar codes were grouped into subcategories, which were then abstracted into broader categories and overarching themes representing the core factors affecting documentation quality. Two researchers independently coded the transcripts, and discrepancies were resolved through discussion and consultation with a third researcher. MAXQDA (version 2022) was used for data management, coding, and visualization of analytical patterns. Reflexive memo writing was employed throughout the analysis to document evolving analytical insights, and constant comparison ensured that both manifest and latent content were considered. In addition, the research team maintained an ongoing reflexive stance by critically examining how their clinical and academic backgrounds in nursing and mental health care could influence coding decisions and theme development. These reflections were systematically recorded and revisited during analytical meetings to minimize interpretive bias.

### Trustworthiness

We applied Lincoln and Guba’s criteria of credibility, transferability, dependability, and confirmability [25]. Credibility was enhanced through prolonged engagement with participants, iterative interviews, and member checking, during which preliminary interpretations were shared with participants for validation. Transferability was addressed by providing rich, contextual descriptions of hospital settings, participant characteristics, and contextual factors. Dependability was ensured through a comprehensive audit trail that included interview records, coding decisions, field notes, and analytical memos. Confirmability was established via reflexive documentation of researchers’ assumptions, independent coding by multiple team members, and review of thematic structures by two external qualitative research experts. To further reinforce confirmability and reflexivity, the research team explicitly articulated their positionality as healthcare researchers with prior experience in nursing and mental health services. Potential preconceptions concerning documentation practices and patient safety were acknowledged from the outset and revisited continuously throughout data collection and analysis. Given the cultural sensitivity of suicide in Iran, researchers were especially attentive to how their professional backgrounds and underlying assumptions might influence interview dynamics-particularly participants’ willingness to disclose challenges related to documentation or legal concerns. To mitigate this, interviewers maintained a neutral, non-judgmental stance, reassured participants of confidentiality, and encouraged open dialogue. Reflexive discussions were held regularly to critically examine how researchers’ perspectives might shape data interpretation and to ensure that participants’ voices remained central to the analysis. These ongoing discussions enhanced transparency throughout the analytic process. Collectively, these measures strengthened the reliability and validity of the findings.

### Ethics approval and consent to participate

This study was approved by the Jiroft University of Medical Sciences Ethics Committee (approval ID: IR.JMU.REC.1402.004) and was conducted in accordance with the principles of the Declaration of Helsinki. All procedures were performed in compliance with relevant guidelines and regulations. Written informed consent was obtained from all participants and/or their legal guardians. One of the authors (Hedyeh Askarpour) is a member of the institutional Ethics Committee; however, in line with the university’s conflict-of-interest policy, she was excluded from all review, deliberation, and decision-making processes concerning the ethical approval of this study. The study was evaluated and approved by the remaining independent committee members.

## Results

This qualitative study included a total of 25 participants: 18 nurses, 5 head nurses, and 2 clinical supervisors, all of whom directly involved in the care of patients with suicide attempts in Iranian hospitals. The sample included 14 females and 11 males, with ages ranging from 25 to 57 years. Clinical experience ranged from 2 to 30 years (mean = 12.3 years). This diversity provided a rich dataset that reflected multiple perspectives across different levels of clinical responsibility and experience (Table 1).

**Table 1:** Demographic characteristics of participants (N = 25).

Characteristic	Category	Frequency (n)
**Gender**	Male	11
	Female	14
**Age Group**	25-35 years	8
	36-45 years	10
	46-60 years	7
**Professional Role**	Nurse (N)	18
	Head Nurse (HN)	5
	Clinical Supervisor (CS)	2
**Affiliated University**	Tehran University of Medical Sciences	7
	Shiraz University of Medical Sciences	5
	Isfahan University of Medical Sciences	5
	Mashhad University of Medical Sciences	4
	Kerman University of Medical Sciences	4
**Years of Experience**	2-5 years	5
	6-15 years	12
	16-30 years	8

Analysis of the interview data identified four overarching categories, eight main themes, and 38 sub-themes related to factors influencing the quality of nursing documentation in suicide care. The four categories were: [1] contextual and organizational inputs, [2] capacity-building processes, [3] interpersonal and team mechanisms, and [4] individual professional mechanisms. An overview of the categories, themes, sub-themes, and representative quotations are presented in Table 2.

**Table 2:** Main categories, themes and sub-themes of factors influencing the quality of nursing documentation in suicide cases.

Categories	Main Themes	Sub-Themes
**Contextual and Organizational Inputs**	Work Environment and Organizational Support	Supportive leadership
		Positive institutional culture
		Clear policies
		Availability of standardized documentation forms
	Staffing and Workload Balance	Adequate staffing
		Balanced patient assignments
		Scheduling support
	Use of Technology and Resources	Electronic health records
		Access to guidelines and protocols
		Digital tools for documentation
		Reference materials and libraries
		Integration of international digital resources
**Capacity -building processes**	Training and Education	Workshops on psychiatric documentation
		In-service training
		Simulation-based practice
		Guidelines updates
		Peer learning sessions
	Supervision and Feedback	Mentorship quality
		Constructive feedback
		Performance reviews
		Continuous guidance
		Encouragement of reflective practice
		Support in complex cases
**Interpersonal and Team Mechanisms**	Communication and Collaboration	Team collaboration
		Respectful interactions
		Peer consultation
		Multidisciplinary interaction
		Conflict resolution mechanisms
**Individual Professional Mechanisms**	Knowledge and Competence	Clinical expertise in mental health
		Familiarity with suicide risk assessment
		Knowledge of documentation standards
		Ethical judgment
		Decision-making skills
		Time management skills
	Motivation and Accountability	Professional responsibility
		Intrinsic motivation
		Accountability
		Commitment to patient safety

The first category, “contextual and organizational inputs”, included three themes: [1] work environment and organizational support, [2] staffing and workload balance, and [3] use of technology and resources.

### Theme 1. Work Environment and Organizational Support

Work environment and organizational support emerged as a prominent organizational influencing the quality of nursing documentation. Participants identified supportive leadership, a positive institutional culture, clear documentation policies, and the availability of standardized documentation forms as key facilitators of comprehensive documentation.

One nurse described the importance of managerial support as follows:

“*Managers who value thorough documentation encourage us to be diligent in our records, since their support signals that accurate recording is recognized as an important part of patient care rather than just paperwork”* (P8).

Similarly, another participant emphasized the influence of organizational culture:

“*A culture that prioritizes patient safety makes us more committed to accurate documentation, as we feel that our work directly contributes to protecting lives and preventing harm”* (P14).

The value of standardized documentation procedures was also highlighted. As one participant noted:

“Having *standardized forms and clear guidelines helps me avoid missing important details, because they serve as a roadmap and remind me of essential points that must always be included”* (P12).

### Theme 2. Staffing and Workload Balance

Staffing and workload balance were consistently identified as important determinants of documentation quality. Participants cited adequate staffing, balanced patient assignments, and appropriate work scheduling as factors that enabled more complete and timely documentation.

One participant described how adequate staffing supported careful documentation:

“*When there are enough nurses in the unit, I can document patients’ information carefully without feeling rushed, which helps me ensure that no detail is overlooked and that the records truly reflect the patient’s condition”* (P5).

Another participant associated balanced workloads with better documentation quality:

“*Reasonable patient loads give me time to document accurately, instead of rushing through notes and potentially overlooking important aspects”* (P19).

The importance of structured work schedules was also emphasized. One nurse explained:

“*Structured shifts allow dedicated time for recording patient information, which ensures that documentation is not pushed aside during busy periods”* (P11).

### Theme 3. Use of Technology and Resources

Access to technology and documentation resources was frequently cited as facilitating accurate and consistent documentation. Electronic health records, digital documentation tools, clinical guidelines, and reference materials were identified as valuable resources that supported documentation practice.

One participant described the role of electronic documentation systems:

“*Digital templates guide me and reduce errors, as they standardize the process and prompt me to include all necessary information”* (P18).

Another participant emphasized the importance of having easy access to documentation resources:

“*Having resources at hand ensures accuracy, whether it’s access to policies, guidelines, or quick-reference materials”* (P23).

The usefulness of digital documentation software was also mentioned. As one nurse explained:

“*Software helps streamline recording and retrieval of information, saving time and reducing the risk of misplacing important details”* (P19).

Evidence-based resources were also viewed as valuable for improving documentation quality. One participant stated:

“*Evidence-based references enhance the quality of documentation, because they allow me to align my notes with scientific standards”* (P10).

The second category, capacity-building processes, consisted of two themes: training and education, and supervision and feedback.

### Theme 4. Training and Education

Training and education were viewed as essential for strengthening documentation skills in suicide care. Workshops, in-service education, simulation-based learning, updated clinical guidelines, and peer learning opportunities were frequently cited as contributing to greater confidence and competence in documentation.

One participant described the impact of formal training on documentation practice:

“*Training sessions improved my confidence in documenting complex cases because I learned practical strategies for handling sensitive and ambiguous situations”* (P10).

The importance of continuous professional education was also emphasized. As one nurse explained:

“*Regular on-the-job training keeps us updated with best practices, reminding us that documentation is a skill that must constantly evolve with new knowledge”* (P13).

Simulation-based learning was identified as another useful educational strategy. One participant commented:

“*Simulated scenarios allowed me to practice recording sensitive cases without pressure, which prepared me for real-life situations where accuracy is critical”* (P1).

Participants also referred to peer learning and updated clinical guidelines as valuable educational resources. Reflecting this view, one participant stated:

“*Learning from colleagues’ experiences enhances my own documentation skills, since practical examples often teach me lessons that books and guidelines cannot”* (P4).

### Theme 5. Supervision and Feedback

Supervision and feedback were described as important mechanisms for improving documentation practice. Mentorship, continuous guidance, regular performance reviews, and reflective discussions were frequently mentioned as supporting documentation quality, particularly in complex suicide cases.

The importance of mentorship was emphasized by one participant, who explained:

“*My mentor guides me on correct documentation practices, offering practical advice that shapes how I write and structure my notes”* (P20).

Constructive feedback was also perceived as an important facilitator of documentation quality. As one participant described:

“*Feedback highlights areas to improve without discouraging me, which helps me grow professionally while maintaining motivation”* (P1).

Several participants referred to the value of supervisory support during complex clinical situations. One nurse stated:

“*Having a supervisor available during difficult cases boosts confidence, as I know I can clarify uncertainties before completing my notes”* (P21).

Reflective practice was another aspect discussed by participants. One participant commented:

“*Reflecting on past cases improves future documentation, since I learn from both my strengths and my mistakes”* (P11).

The third category, interpersonal and team mechanisms, included one theme: communication and collaboration.

### Theme 6. Communication and Collaboration

Communication and collaboration were described as central to comprehensive nursing documentation. Teamwork, respectful professional relationships, peer consultation, multidisciplinary meetings, and conflict resolution mechanisms were identified as facilitators of the information exchange required for documenting suicide care.

The contribution of teamwork to accurate documentation was illustrated by one participant, who stated:

“*Discussing cases with colleagues ensures critical information is not missed, because teamwork often brings out perspectives that I might have overlooked alone”* (P2).

Another participant highlighted the importance of multidisciplinary collaboration:

“*Meetings with doctors, psychologists, and social workers enhance comprehensive record-keeping, since each discipline contributes unique details that enrich the patient’s file”* (P7).

Several participants also described challenges related to inter-professional communication. In particular, nurses reported that documenting their own clinical observations regarding suicide risk could sometimes be challenging especially when these observations differed from those documented by physicians.

One participant explained this challenge as follows:

“*Sometimes we notice warning signs before the physician does, but we hesitate to document them strongly because it might be questioned or not taken seriously”* (P8).

A similar concern was expressed by another participant:

“*If the physician’s note says the patient is stable, it can be difficult for us to document concerns about suicide risk, even if we feel something is not right”* (P15).

In addition, participants described difficulties related to documenting suicide risk in cases where patients concealed or minimized their suicidal intent.

One participant described this situation by stating:

“*Sometimes the patient denies suicidal thoughts, but their behavior suggests otherwise. I’m not always sure how explicitly I should document that, especially when it’s based on my clinical judgment rather than direct statements”* (P6).

Another participant shared a similar experience:

“*In some cases, we are concerned the patient might leave the ward or harm themselves, but documenting this risk can be sensitive, especially if there is no formal confirmation”* (P11).

The fourth category, individual professional mechanisms, consisted of two themes: knowledge and competence, and motivation and accountability.

### Theme 7. Knowledge and Competence

Professional knowledge and clinical competence emerged as essential individual factors influencing documentation quality. Clinical expertise in mental health, familiarity with suicide risk assessment, knowledge of documentation standards, ethical judgment, clinical decision-making, and time management were all cited as contributing to effective documentation.

One participant highlighted the importance of clinical expertise in suicide care:

“*Understanding suicide risk factors allows me to record critical information accurately because I know which warning signs and behaviors must be documented for future reference”* (P3).

The role of structured suicide risk assessment was emphasized by another participant:

“*Knowing the proper assessment tools ensures my documentation is precise and useful, since standardized instruments provide structure and reliability in what I report”* (P7).

Participants also stressed the importance of following documentation standards. As one nurse explained:

“*Following the national documentation guidelines keeps my reports consistent, which is important for ensuring continuity of care across different professionals and units”* (P6).

Ethical considerations were frequently mentioned when documenting suicide-related information. One participant stated:

“*Being aware of ethical principles helps me maintain confidentiality and integrity in my notes, especially when documenting sensitive issues like suicidal behaviors”* (P2).

Clinical decision-making was also identified as an important aspect of documentation. One participant commented:

“*Quick clinical decisions require accurate records to support patient care continuity, since other professionals often rely on my notes to make urgent treatment choices”* (P11).

Time management skills were likewise viewed as important. As one participant noted:

“*Allocating time specifically for notes improves quality, since I can focus without interruptions and reflect more carefully on what needs to be included”* (P6).

### Theme 8. Motivation and Accountability

Motivation and accountability were identified as important personal influences on documentation practice. Professional responsibility, intrinsic motivation, a sense of accountability, and commitment to patient safety were all cited as factors that encouraged participants to document suicide care more carefully and comprehensively.

Professional responsibility was highlighted by one participant, who stated:

“*I double-check my notes because patient safety depends on them, and even small mistakes in documentation can lead to serious consequences”* (P1).

Another participant described documentation as a reflection of professional commitment:

“*I want my documentation to reflect the best care I can provide, so I put effort into making the notes clear, accurate, and professional”* (P14).

Accountability within the organization was also viewed as an important motivator. One participant explained:

“*Knowing my notes are reviewed makes me meticulous, as external oversight motivates me to uphold high standards of accuracy”* (P25).

Participants also emphasized their commitment to patient safety. As one nurse expressed:

“*Every detail in the record can influence care decisions, which makes me more attentive in capturing both major and seemingly minor observations”* (P4).

## Discussion

The present study provides a qualitative understanding of the multilevel factors influencing the quality of nursing documentation in suicide care within Iranian hospitals. Unlike previous studies that primarily relied on documentation audits or quantitative assessments to identify deficiencies [26,27], this study explored nurses’ experiences to better understand why documentation challenges occur in clinical practice. The findings indicate that documentation quality is shaped by the interaction of organizational, interpersonal, and individual professional factors, rather than by isolated influences.

### Contextual and organizational inputs

The findings indicate that organizational conditions provide the foundation for high-quality nursing documentation in suicide care. Participants consistently identified supportive leadership, clear documentation policies, adequate staffing, and access to appropriate documentation resources as essential prerequisites for maintaining accurate records. These findings are consistent with those reported by Molla et al. (2024), who showed that managerial support, resource availability, and organizational commitment positively influence the quality of nursing documentation [28]. Similarly, Pine and Bossen (2020) reported that documentation practices are shaped by institutional structures and organizational workflows rather than individual competence alone [29].

Within the Iranian healthcare context, these organizational factors are particularly significant, as patients following suicide attempts are frequently managed in busy emergency departments or general medical wards, where competing clinical priorities may limit opportunities for comprehensive psychosocial documentation. These findings suggest that strengthening organizational infrastructure—including supportive leadership, standardized suicide-specific documentation procedures, and protected documentation time—may enhance documentation quality and, ultimately, patient safety.

Workload also emerged as an important organizational factor. In line with De Groot et al. (2022), participants described how excessive workload reduced opportunities for comprehensive documentation and increased the likelihood of incomplete records [30]. The present findings further suggest that workload interacts with the design of documentation systems. Therefore, workforce planning should be accompanied by simplified documentation processes and workflow redesign to reduce unnecessary documentation burden while maintaining clinically meaningful records.

Technology was identified as another organizational resource that supported documentation quality. Participants viewed electronic health records, digital documentation tools, and readily accessible clinical guidelines as facilitators of more consistent documentation—a finding consistent with previous evidence demonstrating that electronic documentation systems improve documentation completeness and continuity of care [31]. However, participants also noted that existing electronic health record systems in Iran are largely designed for general medical documentation and do not adequately support suicide-specific assessment. This context-specific finding underscores the need for suicide-focused electronic documentation templates that combine structured risk assessment with sufficient flexibility to record individualized clinical observations and professional judgment.

### Capacity-building Processes

The findings suggest that education, supervision, and feedback are key mechanisms through which documentation quality can be strengthened in suicide care. Participants described workshops, simulation-based learning, peer learning, and constructive feedback as improving their confidence in documenting sensitive and uncertain clinical situations. Similar findings were reported by Hong et al. (2022), who demonstrated that simulation-based educational interventions improve nurses’ confidence and documentation accuracy in complex clinical situations [32]. Likewise, Hebeshy et al. (2025) reported that structured educational programs enhance nurses’ adherence to documentation standards and reduce documentation errors in high-risk care environments [33]. Collectively, these findings reinforce the importance of continuous professional development in maintaining documentation competence.

The present findings further indicate that the effectiveness of educational interventions depends on their relevance to clinical practice. Participants consistently valued practical workshops, simulation exercises, peer learning, and updated clinical guidance, suggesting that experiential learning better prepares nurses to document complex suicide-related situations than theoretical instruction alone. In the Iranian healthcare context, where suicide-specific documentation training remains limited in many hospitals, incorporating realistic case scenarios into continuing professional development programs may strengthen both documentation accuracy and nurses’ confidence in applying clinical judgment.

Supportive supervision and constructive feedback were also identified as important contributors to documentation quality. Consistent with Shahzeydi et al. (2024), who reported that structured clinical supervision improves adherence to professional standards and clinical performance among nursing students [34], participants described mentorship and regular feedback as opportunities to clarify documentation challenges, strengthen professional confidence, and promote reflective learning. These findings suggest that educational initiatives are likely to be most effective when combined with supportive supervision rather than implemented as stand-alone training programs. Integrating simulation-based suicide documentation training, structured mentorship, and multidisciplinary case discussions into continuing professional development may therefore enhance documentation quality and improve patient safety.

### Interpersonal and Team Mechanisms

The findings demonstrate that communication and collaboration are central to high-quality nursing documentation in suicide care. Participants described respectful teamwork, multidisciplinary communication, and peer consultation as facilitators of the exchange of sensitive clinical information and as supports for more comprehensive documentation. These findings are consistent with those of Molla et al. (2024), who reported that collaborative organizational cultures improve documentation quality through enhanced communication and professional support [28]. Similarly, Bunting and Klerk (2022) found that effective interdisciplinary collaboration strengthens the completeness and reliability of nursing documentation in complex clinical settings [35].

A notable contribution of the present study is the identification of professional hierarchy as a contextual influence on documentation practices. Participants described hesitating to document independent nursing concerns when these differed from physicians’ assessments, particularly in relation to suicide risk. This finding suggests that documentation quality may be influenced not only by professional competence or organizational policies but also by power dynamics within multidisciplinary teams. In high-risk situations, reluctance to document independent clinical observations may delay the communication of important concerns and compromise continuity of care.

Participants also highlighted the challenges of documenting suicide risk under conditions of clinical uncertainty, particularly when patients denied suicidal intent despite displaying behaviors suggestive of ongoing risk. Although previous studies have acknowledged the complexity of suicide risk assessment [36], limited attention has been given to how such uncertainty affects nursing documentation. The present findings extend existing knowledge by demonstrating that clinical uncertainty may influence the completeness and clarity of documentation, particularly when nurses must balance professional judgment with ethical and legal considerations.

These interpersonal challenges may be particularly relevant within the Iranian healthcare context, where cultural sensitivity surrounding suicide and hierarchical decision-making structures may encourage cautious documentation practices. The findings suggest that standardized documentation protocols alone are unlikely to be sufficient. Promoting psychologically safe multidisciplinary environments, together with shared documentation training and regular interdisciplinary case discussions, may support more accurate documentation and strengthen patient safety in suicide care.

### Individual professional mechanisms

Professional knowledge, clinical competence, and motivation emerged as important individual determinants of documentation quality in suicide care. Participants’ experiences indicate that accurate documentation requires more than technical recording skills; it depends on nurses’ ability to integrate mental health knowledge, suicide risk assessment, ethical reasoning, and clinical judgment when documenting complex and uncertain situations. Similar findings have been reported by Mabunda et al. (2025), who identified professional competence as a major determinant of documentation quality in mental health settings [17]. Likewise, Kusheba and Mulvihill (2018) emphasized that ethical responsibility and sound clinical reasoning are fundamental to providing safe care for individuals at risk of suicide [36]. These findings reinforce the importance of viewing documentation competence as a professional capability that requires continuous development rather than a skill acquired only during initial nursing education.

A notable contribution of the present study is that nurses described documentation as an active clinical decision-making process rather than a routine administrative task. Particularly when suicide risk was ambiguous or patients minimized their suicidal intent, documentation relied heavily on professional judgment. This finding extends previous research by highlighting that high-quality suicide documentation requires the integration of technical competence with contextual clinical reasoning and ethical decision-making. Strengthening these competencies through case-based education and reflective learning may therefore improve documentation quality in high-risk mental health settings.

Motivation and professional accountability further influenced documentation practices. In line with the findings of Wahyuni et al. (2023), motivated nurses demonstrated greater commitment to documentation standards and patient safety [37]. However, the present findings suggest that motivation is closely shaped by the organizational environment. Supportive leadership, constructive feedback, and recognition appeared to encourage thorough documentation, whereas concerns about criticism or legal consequences sometimes promoted more cautious recording practices. This suggests that documentation improvement strategies should promote accountability while simultaneously fostering supportive organizational cultures that encourage professional autonomy.

The findings also highlight the medico-legal importance of nursing documentation in suicide care. Beyond supporting clinical communication, documentation serves as an essential legal record during adverse event reviews and forensic investigations. Participants’ accounts suggest that concerns regarding legal accountability may influence documentation practices, particularly in situations characterized by clinical uncertainty. This finding emphasizes the need for clear institutional guidance that supports accurate, objective, and legally sound documentation while protecting nurses’ professional judgment. Such guidance may ultimately strengthen patient safety, professional confidence, and the quality of suicide prevention services.

Overall, this study advances current understanding of nursing documentation in suicide care by demonstrating that documentation quality is shaped by the interaction of organizational conditions, capacity-building processes, interpersonal relationships, and individual professional attributes. The findings also highlight professional hierarchy, clinical uncertainty, and the cultural sensitivity surrounding suicide as contextual influences that have received limited attention in previous research. Together, these insights help address the knowledge gap identified at the outset of this study by explaining why documentation challenges persist in everyday clinical practice and suggesting that sustainable improvements require integrated, context-sensitive strategies that combine organizational support, continuing professional education, psychologically safe teamwork, and suicide-specific documentation systems. Although the findings are grounded in the Iranian healthcare context, many of the underlying mechanisms may also be relevant to other healthcare systems, while requiring adaptation to local organizational, cultural, and legal contexts.

## Conclusion

This study demonstrates that the quality of nursing documentation in suicide care is shaped by the interaction of organizational, interpersonal, and individual professional factors rather than by isolated influences. The findings emphasize that improving documentation requires comprehensive, system-level strategies that combine supportive organizational environments, adequate staffing, suicide-specific education, effective supervision, collaborative multidisciplinary practice, and strong professional competence. By providing qualitative insight into the contextual factors influencing documentation practices in Iranian hospitals, this study offers evidence to inform documentation standards, nursing education, and patient safety initiatives. Developing context-sensitive interventions that integrate clinical, ethical, and medico-legal considerations may contribute to more consistent documentation practices and ultimately enhance the quality and safety of suicide care.

### Policy implications and recommendations

The findings of this study suggest that improving nursing documentation in suicide care requires coordinated policy actions at the national, organizational, educational, and technological levels. Given that documentation quality is shaped by the interaction of system-level and professional factors, isolated interventions are unlikely to yield sustainable improvements. Instead, comprehensive strategies that simultaneously strengthen organizational infrastructure, professional competence, and multidisciplinary collaboration are needed to support high-quality documentation in suicide care.

At the national level, the Ministry of Health and Medical Education (MOHME) could consider developing a standardized National Suicide Documentation Protocol as part of Iran’s national nursing documentation framework. Such a protocol could establish minimum documentation standards for suicide-related care, including the structured recording of suicide risk assessments, patients’ disclosures, behavioral warning signs, clinical judgment under conditions of uncertainty, and clear differentiation between nurses’ independent observations and physician-confirmed assessments. Integrating clinical, ethical, and medico-legal guidance within a unified protocol may reduce variability in documentation practices and improve consistency across healthcare settings.

At the organizational level, hospitals should strengthen the structural conditions that support high-quality documentation. This includes ensuring adequate staffing, providing protected time for documentation, promoting supportive leadership, and establishing multidisciplinary documentation review processes involving nursing supervisors, psychiatrists, emergency physicians, and clinical risk managers. Regular audits and constructive feedback should be used primarily as quality improvement strategies rather than punitive monitoring mechanisms, thereby fostering psychological safety and encouraging accurate documentation of suicide-related clinical observations.

Educational and technological strategies should complement these organizational initiatives. Competency-based continuing education programs, particularly those incorporating simulation-based training and case-based learning focused on suicide documentation, may enhance nurses’ confidence and documentation competence in high-risk situations. In parallel, electronic health record systems should be further developed to incorporate suicide-specific documentation templates, structured suicide risk assessment fields, automated reassessment prompts, and clinical decision-support tools. Aligning technological systems with clinical workflows may reduce documentation burden while improving the completeness, consistency, and clinical utility of nursing documentation. Collectively, these policy directions may contribute to strengthening patient safety, improving interdisciplinary communication, and supporting more effective suicide prevention within hospital settings.

### Limitations

This study was conducted in hospitals in Iran, which may limit transferability to other countries with different cultural, policy, and resource contexts. The qualitative design inherently involves subjectivity, and participants’ perspectives may have been influenced by local organizational culture. Additionally, as data were collected through interviews, participants may have been subject to social desirability bias, leading them to emphasize positive practices or underreport challenges related to nursing documentation in suicide care. Importantly, this bias may have had a particular impact on the interpretation of organizational themes such as “supportive leadership” and “work environment.” Given the hierarchical structure of hospital settings in Iran, participants may have been reluctant to openly criticize supervisors or institutional policies, which could have resulted in an overemphasis on positive leadership behaviors and underreporting of managerial shortcomings, communication barriers, or punitive supervisory practices. Therefore, the findings related to organizational support should be interpreted with caution, as they may partially reflect perceived expectations or professional caution rather than fully unfiltered experiences. The study also did not include a longitudinal assessment, which could have provided insights into the sustainability of improvements in documentation practices over time.

### Suggestions for future research

The present findings provide several directions for future research. First, multicenter studies involving different regions of Iran and other low- and middle-income countries are needed to examine how organizational, cultural, and legal contexts influence nursing documentation in suicide care. Comparative cross-cultural studies may help determine which of the identified determinants are context-specific and which are transferable across healthcare systems. Second, longitudinal and implementation studies should evaluate the effectiveness of interventions informed by the present findings, such as suicide-specific documentation protocols, simulation-based educational programs, structured mentorship, and electronic documentation systems incorporating suicide risk assessment tools. Such studies would provide evidence regarding the sustainability of improvements in documentation quality over time. Third, mixed-methods research combining qualitative exploration with quantitative outcome measures could further clarify the relationships between documentation quality, patient safety, continuity of care, and suicide prevention outcomes. Finally, future studies should extend the range of participants by including psychiatrists, emergency physicians, psychologists, hospital managers, and, where ethically appropriate, patients and family caregivers, to obtain a more comprehensive understanding of documentation practices and their implications for multidisciplinary suicide care.

## Supporting information

S1 File Semi-structured interview guide developed by the research team based on a review of international and national literature on nursing documentation and suicide care. (DOCX)
