# Supplementary material for: Exploring multilevel determinants of nursing documentation quality in suicide care: A qualitative Study in Iranian hospitals
Source: PLoS One. 2026 Sep 2;21(9):e0356460. doi: 10.1371/journal.pone.0356460 (PMC13537574; doi:10.1371/journal.pone.0356460)
Supplement: Supplementary file 1 [file pone.0356460.s001.docx]

# Interview Guide: Exploring Multilevel Determinants of Nursing Documentation Quality in Suicide Care: A Qualitative Study in Iranian Hospitals

***Introduction***

1. Thank you for agreeing to participate in this interview.

2. The purpose of this study is to explore factors influencing the quality of nursing documentation in suicide cases.

3. Your participation is voluntary, and you may skip any question or withdraw at any time.

4. With your permission, I will record the interview to ensure accuracy.

5. Do you have any questions before we begin?

***Section 1: Background Information***

(to understand context and participant characteristics)

Could you please tell me about your role, department, and years of experience in nursing?

How often do you deal with patients who have attempted suicide?

What kinds of documentation responsibilities do you typically have in such cases?

***Section 2: Main Questions (Core Topics)***

1. Facilitators of High-Quality Documentation

In your experience, what factors help you document nursing care for suicide patients effectively?

Can you share an example of a situation where documentation was done well and supported patient care?

***2. Barriers and Challenges***

What challenges do you face when documenting care for suicide patients?

Are there specific conditions (workload, organizational policies, emotional stress, etc.) that make documentation more difficult?

***3. Professional Knowledge & Training***

How does your knowledge or training influence your documentation practices in suicide cases?

Do you feel you have received adequate training for documenting such cases? Why or why not?

***4. Organizational and Systemic Factors***

How do hospital policies, supervision, or administrative procedures affect documentation quality?

What role does technology (e.g., electronic health records) play in your documentation process?

***5. Interpersonal and Communication Factors***

How do interactions with colleagues, doctors, or supervisors affect the way you document patient information?

Can you describe a situation where teamwork or communication improved or hindered documentation?

***6. Attitudes and Motivation***

How do personal attitudes, motivation, or emotional responses to suicide cases influence your documentation?

Do you sometimes feel discouraged or hesitant when documenting sensitive cases?

***Section 3: Improvement Suggestions***

What changes or interventions could improve the quality of nursing documentation in suicide cases?

What kind of support (training, supervision, resources, policies) do you think would be most helpful?

***Section 4: Probing Prompts (to deepen responses)***

Could you tell me more about that?

Can you give me an example?

How did that make you feel?

What happened as a result?

***Section 5: Closing***

Is there anything else you would like to add about your experiences with documentation in suicide cases?

Thank you very much for your time and valuable insights.
